# Eco-friendly synthesis for MCM-41 nanoporous materials using the non-reacted reagents in mother liquor

**DOI:** 10.1186/1556-276X-8-120

**Published:** 2013-03-04

**Authors:** Eng-Poh Ng, Jia-Yi Goh, Tau Chuan Ling, Rino R Mukti

**Affiliations:** 1 School of Chemical Sciences, Universiti Sains Malaysia, Minden, 11800, Malaysia; 2 Faculty of Science, University of Malaya, Kuala Lumpur, 50603, Malaysia; 3 Division of Inorganic and Physical Chemistry, Institut Teknologi Bandung, Jl. Ganesha no. 10, Bandung, 40132, Indonesia

**Keywords:** MCM-41, Green synthesis, Mother liquor, Chemical compensation, Chemical waste

## Abstract

Nanoporous materials such as Mobil composite material number 41 (MCM-41) are attractive for applications such as catalysis, adsorption, supports, and carriers. Green synthesis of MCM-41 is particularly appealing because the chemical reagents are useful and valuable. We report on the eco-friendly synthesis of MCM-41 nanoporous materials *via* multi-cycle approach by re-using the non-reacted reagents in supernatant as mother liquor after separating the solid product. This approach was achieved *via* minimal requirement of chemical compensation where additional fresh reactants were added into the mother liquor followed by pH adjustment after each cycle of synthesis. The solid product of each successive batch was collected and characterized while the non-reacted reagents in supernatant can be recovered and re-used to produce subsequent cycle of MCM-41. The multi-cycle synthesis is demonstrated up to three times in this research. This approach suggests a low cost and eco-friendly synthesis of nanoporous material since less waste is discarded after the product has been collected, and in addition, product yield can be maintained at the high level.

## Background

Mobil composite material number 41 (MCM-41) is a mesoporous material that was first discovered in 1992 [1,2]. It has a hexagonal array of uniformly sized one-dimensional mesopores with a pore diameter of 2 to 10 nm. The research on these nanoporous materials is of interest especially in catalysis, adsorption, supports, and carriers due to its excellent properties such as high surface area, high thermal stability, high hydrophobicity, and tunable acidity [3,4]. Furthermore, the pore size of MCM-41 can be tailored by using surfactants with different chain lengths and/or auxiliary structure-directing agent [5,6].

Several methods such as hydrothermal and solvothermal treatments have been used for the synthesis of MCM-41 meso-ordered material [7-9]. The concept of all these approaches is to terminate the synthesis process after the synthesis is complete; the nanomaterials are formed in sol suspensions and are recovered by filtration or centrifugation. The remaining synthesis solution is usually discarded after the nanoporous materials are collected. However, these conventional methods bring several drawbacks to the environment and industry. For instance, large amounts of initial reactants which remain unused in the remaining solution, including the expensive organic surfactant template, silica and corrosive solvent such as NaOH, is discarded during the recovering of mesostructured particles. This causes the synthesis of nanoporous material an uneconomical process; it is not cost effective for chemical industries. Moreover, the disposal of unused chemical reagents especially the surfactant template after the synthesis results in severe health hazard and adverse environmental effect [10,11]. Thus, any new insight regarding the replacing, recycling, or reusing of the valuable chemicals in the synthesis of nanoporous materials is highly appreciated.

Recently, the use of electronic (e-waste) [12] and natural wastes such as coal fly ash [13-17] and rice husk ash [18] as silica sources for the preparation of MCM-41 has been reported. In general, the ashes and electronic resin waste are treated with sodium hydroxide to extract the silica out before their introduction into the MCM-41 synthesis solution. With this strategy, the inorganic waste is re-used, and it can be converted into more valuable and useful materials which may have important economic implications. In the environmental aspect, converting silica waste into nanoporous materials such as MCM-41 may provide another way for preserving the environment.

Although eco-friendly synthesis on MCM-41 using natural wastes has been reported to date, there is no study on the synthesis of MCM-41 by recycling the mother liquid. One of the reasons is that the change in the molar composition and the pH of the precursor solution will have a profound impact on the resulting materials, i.e., no solid product, amorphous, new or mixture of two mesophases (lamellar, cubic, disordered) will be formed instead of the desired single hexagonal mesophase [2]. In this work, MCM-41 is prepared with a green synthesis strategy by reusing non-reacted reagents remaining in the synthesis solution followed by supplementary compensation of the consumed chemicals and pH adjustment. The chemical compositions of mother liquor and solid product of each cycle were then characterized by using dry solid mass analysis, thermogravimetry (TG)/differential thermal analysis (DTA), X-ray diffraction (XRD), Fourier transform infrared spectroscopy (FTIR), ^29^Si magic-angle-spinning (MAS) solid-state nuclear magnetic resonance (NMR), transmission electron microscopy (TEM), atomic absorption spectrometry (AAS) and N_2_ adsorption-desorption analyses.

## Methods

### Multi-cycle synthesis of MCM-41

**Figure 1:**
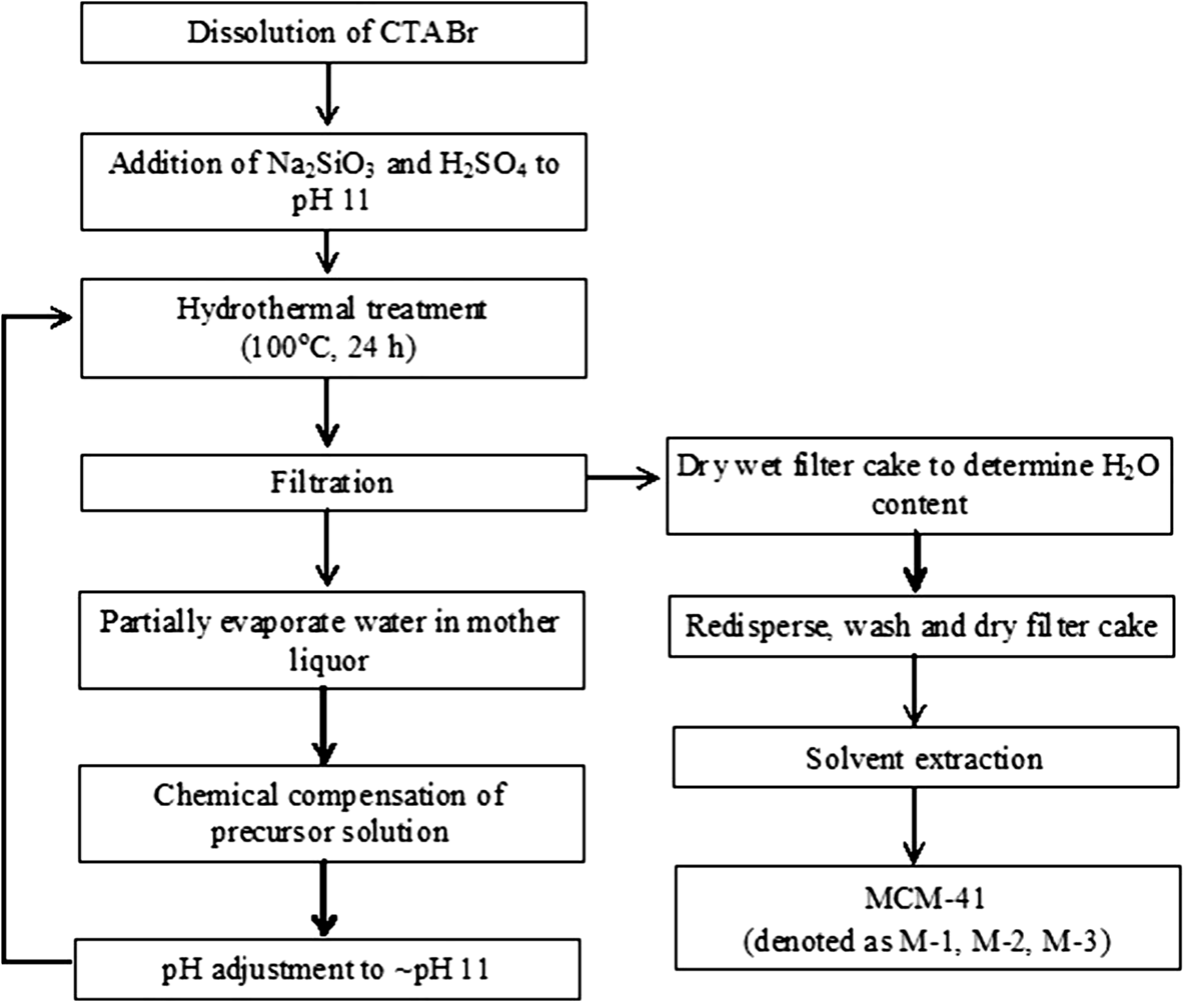
Flow diagram of multi-cycle synthesis of MCM-41 materials.

The nanoporous MCM-41 powder was synthesized from an alkaline solution containing cetyltrimethylammonium bromide (CTABr, 98%; Sigma-Aldrich, St. Louis, MO, USA), sodium silicate solution (8% Na2O, 27% SiO2; Merck & Co., Inc., Whitehouse Station, NJ, USA), H_2_SO_4_ (97%; Merck & Co., Inc., Whitehouse Station, NJ, USA), and distilled water. Typically, CTABr (5.772 g) was first dissolved in a 125-mL polypropylene bottle containing distilled water (79.916 g) under stirring (Figure 1). Sodium silicate (21.206 g) was then introduced into the mixture before H_2_SO_4_ (1.679 g) was added dropwise to give a solution with a pH of 11.0 and a composition molar ratio of 1 CTABr/1.76 Na_2_O/6.14 SiO_2_/335.23 H_2_O. The mixture was allowed to heat in an oven at 100°C for 24 h.

The mother liquor was separated *via* filtration, and the water from the filtrate was partially evaporated at 55°C for 16 h to enable compensation analysis. For the MCM-41 wet filter cake on clay filter, the mass of water in it was estimated by measuring the mass of the solid before and after drying at 60°C for 14 h. The dried solid was then allowed to redisperse again in water, and the solid product was purified by washing with distilled water until the pH of the solid became 7.0. The purified solid was dried at 80°C overnight, and the mass of purified solid was measured again.

Prior to the second and third synthesis cycles, the chemical composition of the non-reacted solutions was analyzed (please refer to the ‘Characterization’ subsection) and was adjusted to the original one by adding the required amount of CTABr, sodium silicate, and water. The H_2_SO_4_ was then added slowly under stirring until a pH of approximately 11.0 was reached using a pH meter (Ohaus Starter 3000, Parsippany, NJ, USA) to monitor the pH of the solution. The MCM-41 nanoporous materials prepared from the first, second, and third synthesis cycles will be denoted as M-1, M-2, and M-3, respectively.

The organic template in the as-synthesized MCM-41 was removed and recovered through extraction by refluxing the solid (1.5 g) in 1 M hydrobromic acid ethanolic solution (500 mL) at 75°C for 24 h. The template-free MCM-41 was filtered, washed with ethanol, and dried for 10 h at 100°C in vacuum [19]. On the other hand, the ethanol in the filtrate solution was distilled out at 80°C, and the surfactant was recrystallized in a mixture solution of acetone/ethanol (95:5 in volume) after the acid in the solution was neutralized [20]. The recrystallized CTABr white solid was purified with ethanol and dried at 70°C overnight.

### Characterization

X-ray powder diffraction patterns were recorded using a Siemens D5000 Kristalloflex diffractometer (Munich, Germany) with a monochromated Cu Kα radiation in the angular range from 1.7° to 10° (2*θ*) with a scanning speed of 0.02°·s^−1^. TEM was performed using a Philips CM-12 microscope (Amsterdam, The Netherlands) with an accelerating voltage of 300 kV. The silica content was measured using an AAnalyst 200 atomic absorption spectrometer (PerkinElmer, Waltham, MA, USA). The organic template moiety in the sample was determined using a Mettler TGA SDTA851 instrument (Mettler-Toledo, Columbus, OH, USA) with a heating rate of 10°C·min^−1^ under nitrogen flow. Nitrogen adsorption-desorption analysis was conducted using a Micromeritics ASAP 2010 instrument (Norcross, GA, USA). The template-free sample was first degassed at 250°C for 3 h followed by nitrogen adsorption measurement at −196°C. The surface physicochemical properties were then calculated using the Brunauer-Emmett-Teller (BET) and the Barrett-Joyner-Halenda (BJH) models [21]. Solid-state ^29^Si-MAS-NMR spectra were recorded using a Bruker Ultrashield 300 spectrometer (Madison, WI, USA) operating at 300 MHz with tetramethylsilane as a reference. The measurement was carried out at 79.4 MHz and single-contact cross-polarization pulse program was used. The spectra were acquired with a pulse length of 2.7 μs, a repetition time of 6 s, and a contact time of 4 ms. The FTIR spectra of the as-synthesized solid products were obtained with a PerkinElmer spectrometer (System 2000) using the KBr pellet technique (KBr/sample weight ratio = 150:1).

## Results and discussion

The chemical composition of the initial and re-used solutions characterized by dry mass, AAS, and TG/DTA analyses is summarized in Table 1. As can be seen, large amounts of silicate solution (approximately 15 g) and CTABr (approximately 3.5 g) were consumed for three subsequent synthesis cycles of MCM-41. Initially, the CTABr was dissolved in distilled water, and silica was precipitated out after sodium silicate was added into the CTABr solution. At this stage, silicate oligomers act as multidentate ligands with high charge density at head groups, which leads to a lamellar organization of the surfactant [22]. As the acid is introduced, polycondensation and polymerization of silica take place, resulting in the dissolution of lamellar phase. At pH close to 11.0, this dissolution is followed by the formation of the hexagonal MCM-41 material [22,23].

**Table 1:** Compensated chemicals added into non-reacted mother liquor for MCM-41 synthesis cycles and MCM-41 solid yield

**MCM-41 synthesis**	**1st cycle**	**2nd cycle**	**3rd cycle**
Non-reacted mother liquor (g)	0	54.404^a^	63.337^a^
**Added reagents**			
Na_2_SiO_3_ (g)	21.206	15.664	15.560
CTABr (g)	5.772	3.750	3.251
H_2_O (g)	79.916	31.882	27.110
H_2_SO_4_ (g)	0.603	2.082	0.9881
pH	10.78	10.80	10.80
Solid yield, gram (wt.%)^b^	8.034 g (73.6%)	7.851 (71.9%)	7.694 (78.3%)

^a^After evaporating water at 55°C for 16 h.

^b^$\mathrm{Solid}\;\mathrm{yield}\left(\%\right)=\frac{\mathrm{Weight}\;\mathrm{of}\;\mathrm{calcined}\;\mathrm{solid}\;\mathrm{after}\;\mathrm{water}\;\mathrm{being}\;\mathrm{removed}\;\left(g\right)}{\mathrm{Initial}\;\mathrm{weight}\;\mathrm{of}\;\mathrm{Si}O_{2}\left(g\right)}\times 100\%$.

pH was determined to be the most important of the investigated synthesis parameters in affecting pore ordering and mesophase. The solubility and the rate of dissolution of silica increases with the increasing pH resulted in a decrease of the total interfacial area and a more long-range pore ordering [24,25]. High pH results in fast and complete hydrolysis where polymerization can occur within a few minutes [25]. When the pH of the precursor solution decreased (higher acidity), it was observed that the intensity of diffraction peaks in the XRD pattern decreased, suggesting a considerable decrease of pore ordering.

It is important to note that little to no-solid product was formed in the re-used mother liquor before chemical compensation due to insufficient chemicals present in the precursor solution. Thus, supplementary compensation of the consumed chemicals onto mother liquor and pH adjustment are needed before proceeding to the second cycle of synthesis. One should note that amorphous, lamellar, or cubic phase was obtained as single or mixed products when the chemical composition and the pH of the solution were not correctly adjusted (e.g., template/H_2_O ratio is high).

**Figure 2:**
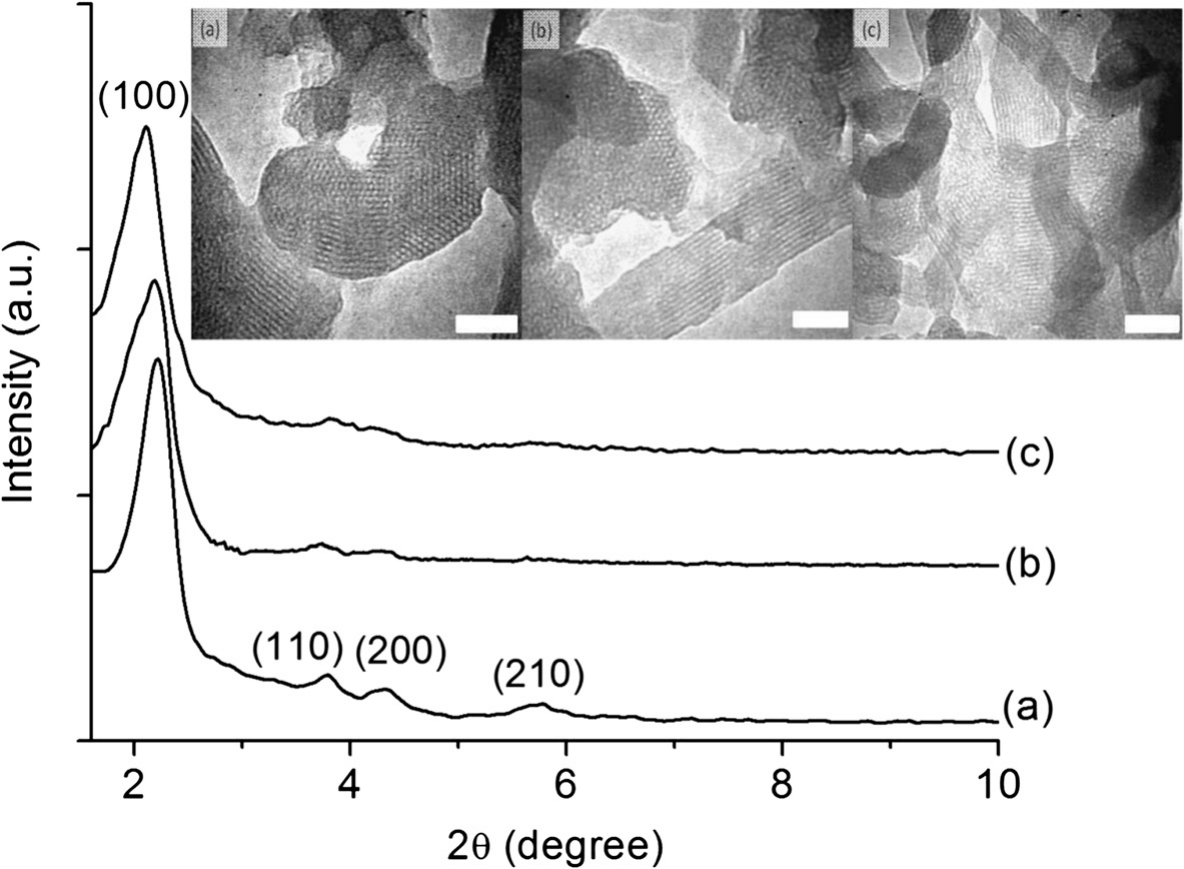
**XRD patterns and TEM images (inset) of as-synthesized MCM-41 nanomaterials synthesized from three subsequent cycles.** (**a**) M-1, (**b**) M-2, and (**c**) M-3. Scale bar = 50 nm.

The ordered mesoporosity of MCM-41 solids for three subsequent cycles is confirmed by XRD analysis (Figure 2). The XRD pattern of all as-synthesized MCM-41 molecular sieves exhibits an intense signal at 2*θ* = 2.2° corresponding to (100) plane and three small signals between 3.5° and 6.0° due to (110), (200), and (210) planes which confirm the presence of well-defined hexagonal MCM-41 [1,2]. Neither lamellar or cubic phase nor amorphous products were observed in the diffractograms, showing that only MCM-41 solids were obtained as pure hexagonal phase after the chemical compositions in the three subsequent synthesis cycles were adjusted to the desired molar ratio and pH. On the other hand, less intense and broadened diffraction peaks were observed for both M-2 and M-3, and this revealed that the ordering degree of both samples slightly decreased in comparison with M-1. Nevertheless, the characteristic diffraction peaks of both samples were retained, indicating that the long-range order of nanoporous hexagonal channels was still preserved after chemical compensation. Also, small peak shifting towards lower diffraction angle was also detected in these two samples which could be explained by a slight increase in the pore size as a result of varied packing of the nanoporous silica particles [25].

The XRD results were further confirmed by TEM analysis. Long-range order of the hexagonal pore arrays could be seen in M-1, and the observation was well agreed with the XRD study (inset of Figure 2a). On the other hand, M-2 and M-3 showed a lower ordering degree than M-1. Nevertheless, the hexagonal periodicity of the mesophase of three MCM-41 samples was basically maintained.

The solid yield of the MCM-41 silica materials for the three subsequent cycles was calculated to be 73.6, 71.9, and 78.3 wt.%, respectively, according to dry mass solid analysis (Table 1). Thus, the solid product yield was considerably high and constant for three subsequent cycles. These results are suggesting that the re-use of the non-reacted precursor solutions is possible as the surfactant template is completely preserved and does not decompose during hydrothermal treatment at mild hydrothermal condition (100°C). Additionally, no organic template and inorganic solution are disposed to the environment, and the chemicals are re-used entirely (CTABr surfactant occluded in MCM-41 framework is extracted out and can be re-used after purification), and thus, this method is revealed as benign to the environment.

**Figure 3:**
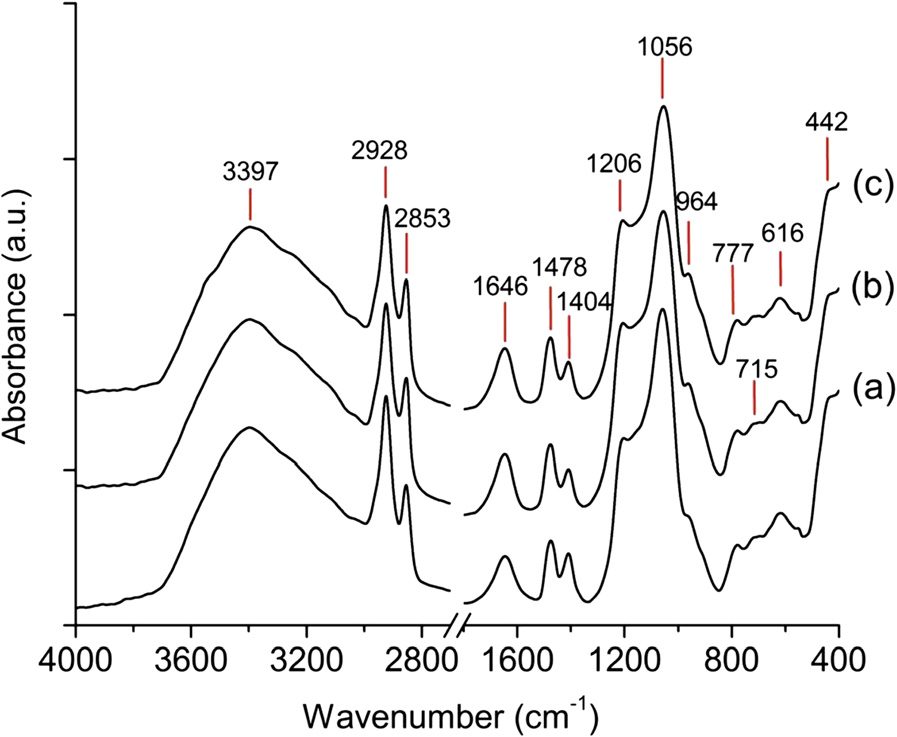
Infrared spectra of as-synthesized samples for three subsequent cycles: (a) M-1, (b) M-2, and (c) M-3.

Figure 3 shows the IR spectra of the three MCM-41 samples. It was observed that the as-synthesized M-1, M-2, and M-3 displayed similar absorption bands. The broad signal at 3,397 cm^−1^ was assigned to water O-H stretching mode, and its bending vibration mode was detected at 1,646 cm^−1^. The presence of absorption bands at 2,928, 2,853, 1,491, 1,478, 1,468, 1,420, 1,404, and 1,377 cm^−1^ was due to the presence of organic template confined in MCM-41 mesopores [26].

In addition, the presence of absorption bands at 1,206 and 1,056 cm^−1^ could be assigned to the asymmetric stretching vibrations of Si-O-Si, while the symmetric stretching vibrations of Si-O-Si resonated at 777 and 616 cm^−1^. Moreover, the IR band at 442 cm^−1^ was attributed to the bending vibration of Si-O-Si. A small signal was also detected at 964 cm^−1^ which was due to the bending mode of surface Si-OH. Low intensity of this signal indicated that only a small amount of silanol group was present in the MCM-41 samples [26].

**Figure 4:**
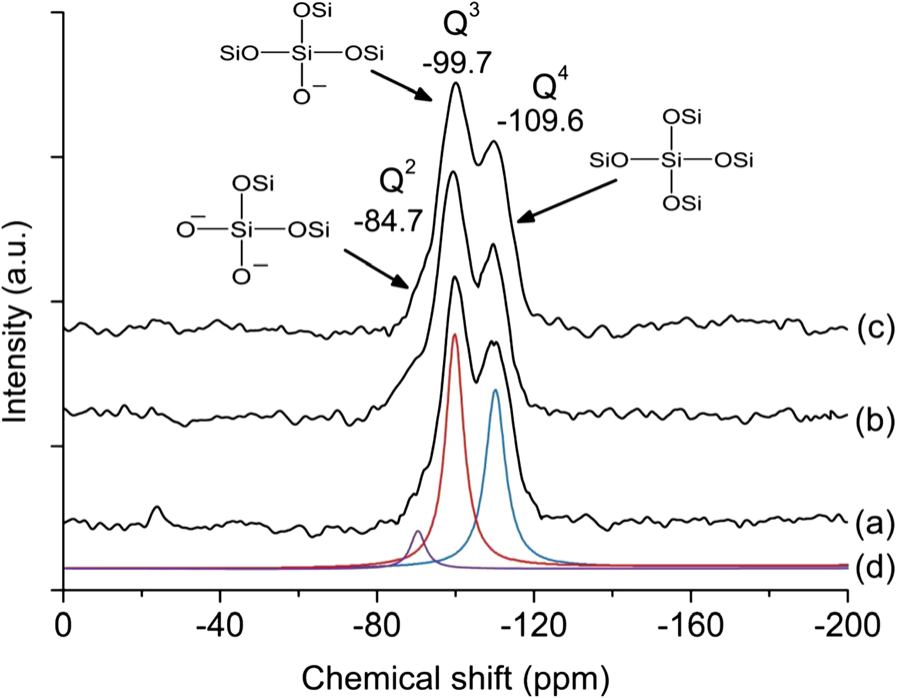
^**29**^**Si MAS NMR spectra of as-synthesized (a) M-1, (b) M-2, (c) M-3, and (d) deconvolution of spectrum M-1.**

A similar conclusion could also be drawn from the ^29^Si MAS NMR spectroscopy. The solid-state ^29^Si-MAS-NMR spectra of M-1, M-2, and M-3 were shown in Figure 4. All samples showed two distinct peaks at −99.7 and −109.6 ppm, which could be assigned as surface vicinal silanol groups (Q^3^) and framework silica (Q^4^), respectively [27]. Furthermore, a weak shoulder was also detected at −84.7 ppm especially for M-2 which was assigned to the surface geminal groups (Q^2^). The relative peak areas of the spectra and the Q^4^/Q^3^ ratio were calculated and were given in Table 2. From the deconvoluted data, M-1 had the highest Q^4^/Q^3^ ratio (0.75), indicating M-1 had the most ordered structure in the nanoporous framework. In contrast, M-2 showed the lowest Q^4^/Q^3^ ratio (0.64) which could be explained by a lower degree of polycondensation of the silicate species. The finding agrees with those determined from the XRD and TEM data (Figure 2b).

**Table 2:** ^**29**^**Si-MAS-NMR deconvolution results**

**Samples**	**Q**^**4 **^**(%)**	**Q**^**3 **^**(%)**	**Q**^**2 **^**(%)**	**Q**^**4**^**/Q**^**3 **^**ratio**
M-1	0.41	0.55	0.04	0.75
M-2	0.35	0.55	0.10	0.64
M-3	0.39	0.53	0.08	0.74

Error of deconvolution: Q^4^, 1%; Q^3^, 5%; Q^2^, 14%.

TG analysis is a powerful analytical technique that can be used to determine the organic components of a material by monitoring the weight loss as the specimen is heated. Using this technique, the amount of organic template is consumed in the synthesis (occluded in as-synthesized MCM-41), thus can be estimated (Note: the quantity of template lost during the purification process of the samples is also taken into account). It was found that the CTA^+^/SiO_2_ molar ratios of M-1, M-2, and M-3 were 0.16, 0.14, and 0.13, respectively, which were in the range of 0.1 to 0.2, a value previously found for a well-organized hexagonal mesophase [25]. From this chemical analysis, it appeared that six to eight SiO^−^ groups compensated one CTA^+^ organic cation.

The TG curves of three as-synthesized samples had a similar shape with slight difference in the percentage of weight loss (please refer to Additional file 1: Figure S1). In the first stage, the weight loss of approximately 6% at below 130°C was attributed to desorption of water. In the second (weight loss of 33% to 38% at 130°C to 340°C) and third (weight loss of approximately 4% at 340°C to 550°C) stages, the weight losses were due to the thermal decomposition of organic template *via* Hofmann elimination [28]. In the fourth stage, at the temperature above 500°C, the weight loss was caused by the condensation of silanol groups to form siloxane bonds [29]. From the TG results, it can be summarized that the MCM-41 nanoporous silica synthesized from three subsequent cycles contained almost the same amount of template (total weight loss of 36 to 41 wt.% in the range of 120°C to 500°C), demonstrating that the consumption of the organic template during the formation of MCM-41 was almost constant in each step of the multi-cycle synthesis.

**Figure 5:**
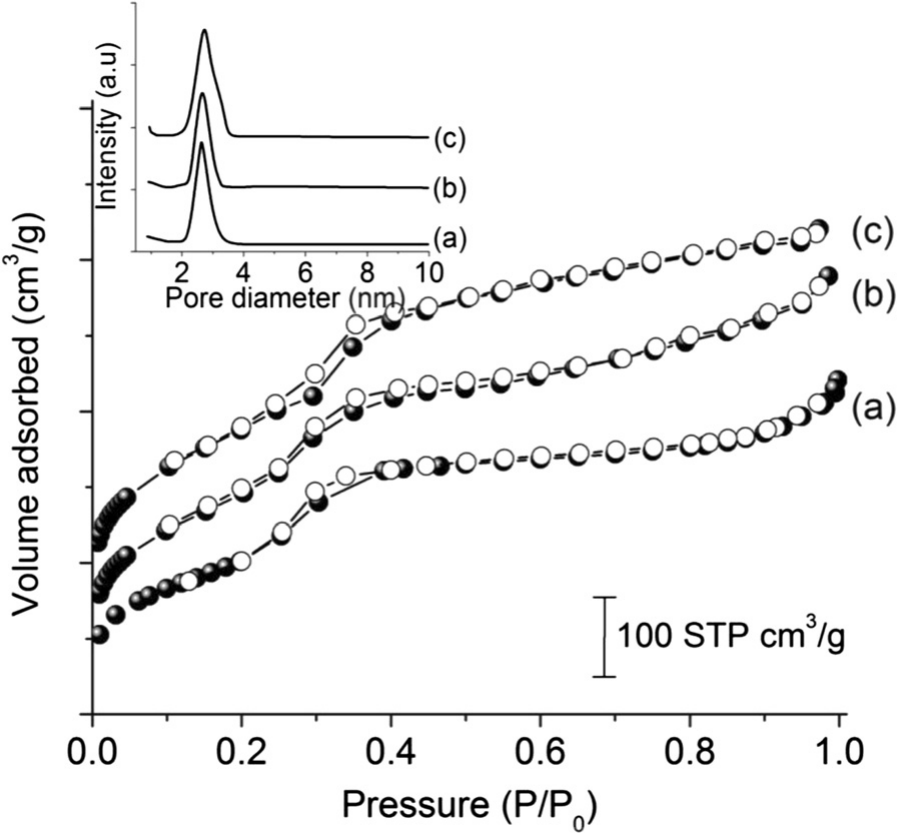
**Nitrogen adsorption-desorption isotherms and BJH pore diameter distribution (inset) of MCM-41 meso-ordered materials synthesized from three subsequent cycles: (a) M-1, (b) M-2 and (c) M-3.** Solid symbols denoted adsorption, and open symbols denoted desorption.

The N_2_ adsorption-desorption isotherms for the MCM-41 nanoporous materials were of type IV with type H1 hybrid loop [30] in accordance with IUPAC classification (Figure 5). A sharp adsorption-desorption step at *P*/*P*_o_ range of 0.3 to 0.35 was observed for all the solids due to pore filling of uniform pores of hexagonal lattice. Table 3 showed that M-1, M-2, and M-3 had high surface areas (above 500 m^2^·g^−1^) and pore volumes (above 0.60 cm^3^·g^−1^), which could be explained by their high degree of ordering. Among the three samples, the M-2 and M-3 possessed higher pore volume over M-1. The difference in the total pore volume of these samples was attributed to the varied packing of the nanoporous silica particles [25]. The pore size distribution of the primary nanopores based on BJH calculation method for M-1, M-2, and M-3 was measured (inset of Figure 5). All samples showed a narrow pore distribution wherein M-3 offered the largest pore size (highest peak centered at 2.72 nm) among the three synthesized samples, and M-1 had the smallest pore size (approximately 2.62 nm).

**Table 3:** Textural properties of the MCM-41 samples

**Samples**	**d**_**100 **_**spacing (nm)**	**Unit cell, a**_**0 **_**(nm)**^**a**^	**Pore size (nm)**^**b**^	**Surface area, *S***_**BET **_**(m**^**2**^**·g**^**−1**^**)**	**Pore volume, *V***_**total **_**(cm**^**3**^**·g**^**−1**^**)**
M-1	3.96	4.57	2.62	544	0.63
M-2	4.03	4.65	2.65	680	0.81
M-3	4.21	4.86	2.72	669	0.80

^a^ a_0_ = 2d_100_/√3.

^b^ Average pore diameter by calculated BJH method.

A scheme representing the total utilization of chemical reagents for conventional one-step and multi-step syntheses of MCM-41 are illustrated in Table 4. The total consumption of reagents is calculated based on five synthesis batches or cycles of MCM-41 nanoporous solid. In the multi-step synthesis approach, it is found that the consumption of reagents can be saved and reduced up to 17.67% and 26.31% for silica source and CTABr surfactant, respectively, in comparison with the conventional single-batch approach. Thus, using multi-cycle synthesis, the synthesis cost, which is one of the major concerns in the industries, is decreased considerably. Furthermore, the chemical waste eliminated to the environment such as organic template and silicate can be decreased up to nearly 90% when multi-cycle synthesis method is employed (not shown).

**Table 4:** Total chemical reagents used for conventional and multi-step syntheses of MCM-41

	**Conventional approach**	**Multi-cycle approach**	**Amount of chemical saved (%)**
*Total chemicals consumed*			
Na_2_SiO_3_ (g)	42.412	34.918	17.67
CTABr (g)	11.543	8.506	26.31
H_2_O (g)	159.832	92.513	42.12

The calculation is based on five synthesis batches or cycles.

Meanwhile, the CTABr in the as-synthesized samples was successfully recovered after solvent extraction using ethanolic solution (please refer to Additional file 1: Figure S2). It was found that the product yield of CTABr after re-crystallization and purification was 84.6%. The regenerated CTABr can be re-used back for the synthesis of MCM-41 which further reduced the cost and consumption of expensive organic template. Furthermore, the ethanol solution used in organic template extraction can be distilled, separated, and re-used without disposing to the environment.

In short, the low consumption of expensive and harmful chemical reagents is demonstrated; thus, large cost saving and environment protection are achieved. Moreover, this method might offer as another green synthesis for other important nanoporous molecular sieves such as SBA-15, MCM-48, chiral mesoporous silica, KIT-1, etc., where the product yield is considerably maintained by re-using the same non-reacted initial reagents, thus decreasing the synthesis cost, making possible the chemical process to be environmentally benign.

## Conclusions

In summary, using a simple multi-cycle method, MCM-41 nanoporous materials can be synthesized in a more eco-friendly and economical way. The obtained samples in three subsequent cycles exhibited remarkable high-BET specific surface area (above 500 m^2^·g^−1^) and high pore volume (above 0.60 cm^3^·g^−1^) while maintaining its well-ordered hexagonal mesostructure. The MCM-41 synthesized from the three subsequent batches had also almost similar degree of ordering, morphology, yield, pore size, and chemical composition.

The multi-cycle synthesis approach in this work is beneficial from the environmental perspective because the amount of waste produced is minimized by recycling synthesis materials which results in environmental problems. This approach is also beneficial in terms of economic perspective as re-use of chemical reactants reduces the production cost in chemical industries.

## Competing interests

The authors declare that they have no competing interests.

## Authors’ contributions

JYG carried out the main experimental work. EPN supervised the research activity and organized the manuscript. JYG and RRM did the chemical characterization. RRM, TCL, and EPN participated in the discussion of results and helped make critical comments in the initial draft of the manuscript. All authors read and approved the final manuscript.

## Authors’ information

JYG is a MSc student of the University Sains Malaysia (USM). EPN is an associate professor at the USM. TCL is a professor at the University of Malaya. RRM is an assistant professor at the Institute Teknologi Bandung.

## Supplementary Material

Additional file 1: Figure S1. TG curves of as-prepared MCM-41 synthesized from three subsequent cycles: (a) M-1, (b) M-2, and (c) M-3. **Figure S2.** Infrared spectra of fresh CTABr (black) and CTABr recrystallized from waste mother liquor (red). The presence of -OH bands at 3,375 and 1,630 cm^−1^ in recrystallized CTABr are due to the adsorption of moisture from environment. (DOCX 91 kb) Click here for file
