# Supplementary material for: Eco-friendly synthesis for MCM-41 nanoporous materials using the non-reacted reagents in mother liquor
Source: Nanoscale Res Lett. 2013 Mar 4;8(1):120. doi: 10.1186/1556-276X-8-120 (PMC3599658; doi:10.1186/1556-276X-8-120)
Supplement: Additional file 1: Figure S1. — TG curves of as-prepared MCM-41 synthesized from three subsequent cycles: (a) M-1, (b) M-2, and (c) M-3. Figure S2. Infrared spectra of fresh CTABr (black) and CTABr recrystallized from waste mother liquor (red). The presence of -OH bands at 3,375 and 1,630 cm−1 in recrystallized CTABr are due to the adsorption of moisture from environment. (DOCX 91 kb) [file 1556-276X-8-120-S1.docx]

**Additional file 1**


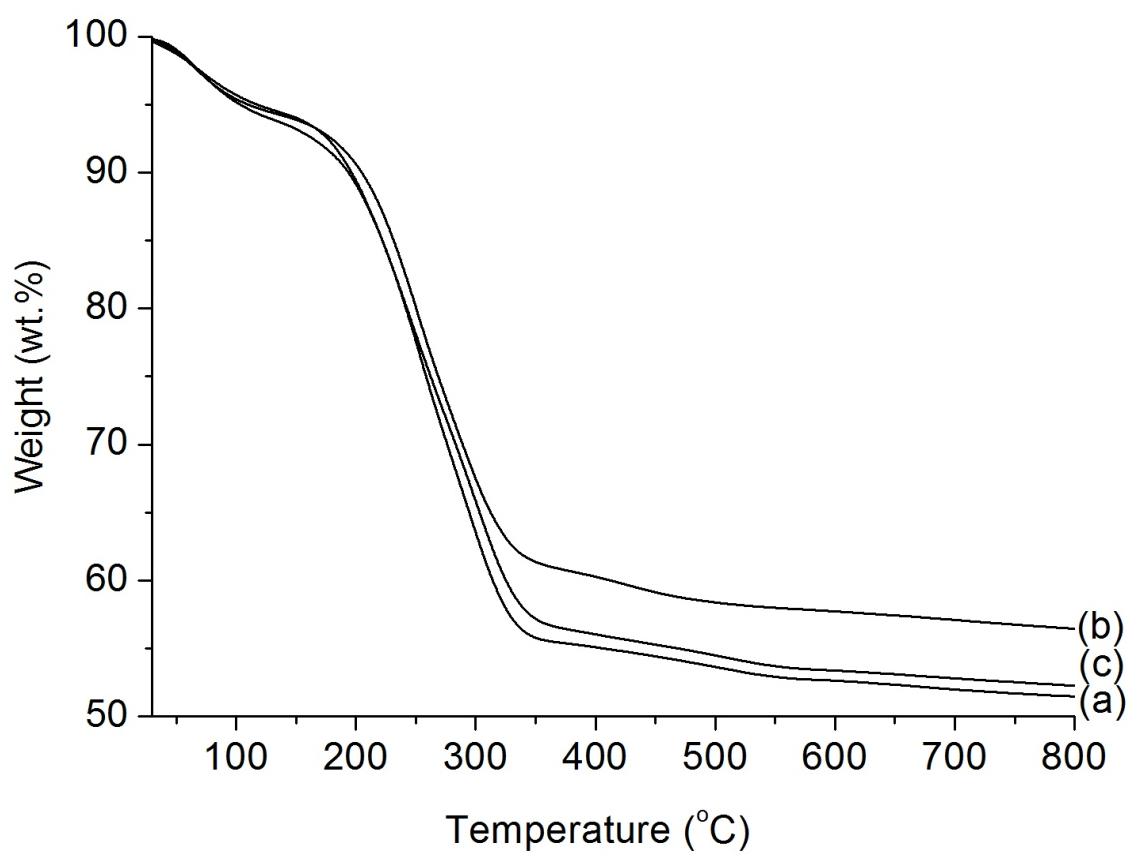


**Figure S1** TG curves of as-prepared MCM-41 synthesized from three subsequent cycles: (a) M-1, (b) M-2 and (c) M-3.

**Figure S2** Infrared spectra of fresh CTABr (black) and CTABr recrystallized from waste mother liquor (red). The presence of -OH bands at 3,375 and 1,630 cm^−1^ in recrystallized CTABr are due to the adsorption of moisture from environment.
